# Supplementary material for: Cytological Samples: An Asset for the Diagnosis and Therapeutic Management of Patients with Lung Cancer
Source: Cells. 2023 Feb 27;12(5):754. doi: 10.3390/cells12050754 (PMC10001120; doi:10.3390/cells12050754)
Supplement: Supplementary file 1 [file cells-12-00754-s001.zip › Table S1.pdf]

**Primary antibody**	**Clone**
Cytokeratin	AE1/AE3/PCK26
Cytokeratin 5/6	D5/16B4
Cytokeratin 7	OV-TL12/30
Cytokeratin 20	KS20.8
Vimentin	V9
Desmin	DE-R-11
Epithelial Membrane Antigen EMA	E29
Epithelial-CAM EpCAM	MOC31
ΔNp63 (p40)	polyclonal rabbit
Calretinin	polyclonal rabbit
WT1	6F-H2
Thyroid Transcription Factor TTF1	8G7G3/1
NKX3.1	polyclonal rabbit
PAX8	MRQ-50
GATA-3	L50-823
CD45	2B11+PD7/26
PS100	polyclonal rabbit
HBM45	HBM45
Melan A	A103
Neuron-Specific Enolase NSE	BBS/NC/V1-H14
Chromogranin A	DAK-A3
Synaptophysin	DAK-SYNAP
CD56 (NCAM)	123C3
Isotype rabbit	Polyclonal rabbit
Isotype mouse	IgG mouse
